# Frame-shift mediated reduction of gain-of-function p53 R273H and deletion of the R273H C-terminus in breast cancer cells result in replication-stress sensitivity

**DOI:** 10.18632/oncotarget.27975

**Published:** 2021-06-08

**Authors:** Viola Ellison, George K. Annor, Clara Freedman, Gu Xiao, Devon Lundine, Elzbieta Freulich, Carol Prives, Jill Bargonetti

**Affiliations:** ^1^The Department of Biological Sciences, Hunter College, City University of New York, New York, NY, USA; ^2^The Graduate Center Biology and Biochemistry Programs, City University of New York, New York, NY, USA; ^3^Department of Biological Sciences, Columbia University, New York, NY, USA; ^4^Department of Cell and Developmental Biology, Weill Cornell Medical College, New York, NY, USA

**Keywords:** mutant p53, gain-of-function, oligomerization, DNA replication, frame-shift

## Abstract

We recently documented that gain-of-function (GOF) mutant p53 (mtp53) R273H in triple negative breast cancer (TNBC) cells interacts with replicating DNA and PARP1. The missense R273H GOF mtp53 has a mutated central DNA binding domain that renders it unable to bind specifically to DNA, but maintains the capacity to interact tightly with chromatin. Both the C-terminal domain (CTD) and oligomerization domain (OD) of GOF mtp53 proteins are intact and it is unclear whether these regions of mtp53 are responsible for chromatin-based DNA replication activities. We generated MDA-MB-468 cells with CRISPR-Cas9 edited versions of the CTD and OD regions of mtp53 R273H. These included a frame-shift mtp53 R273H*f*s387, which depleted mtp53 protein expression; mtp53 R273HΔ381-388, which had a small deletion within the CTD; and mtp53 R273HΔ347-393, which had both the OD and CTD regions truncated. The mtp53 R273HΔ347-393 existed exclusively as monomers and disrupted the chromatin interaction of mtp53 R273H. The CRISPR variants proliferated more slowly than the parental cells and mt53 R273H*f*s387 showed the most extreme phenotype. We uncovered that after thymidine-induced G1/S synchronization, but not hydroxyurea or aphidicholin, R273H*f*s387 cells displayed impairment of S-phase progression while both R273HΔ347-393 and R273HΔ381-388 displayed only moderate impairment. Moreover, reduced chromatin interaction of MCM2 and PCNA in mtp53 depleted R273H*f*s387 cells post thymidine-synchronization revealed delayed kinetics of replisome assembly underscoring the slow S-phase progression. Taken together our findings show that the CTD and OD domains of mtp53 R273H play critical roles in mutant p53 GOF that pertain to processes associated with DNA replication.

## INTRODUCTION

The p53 tumor suppressor protein is well known as a transcription factor but p53 also has transcription independent functions [1]. Interestingly, one of these functions involves regulation of DNA replication. The wild-type (wtp53) protein regulates replication fork progression [2–4]. The direct molecular mechanism of wtp53 during DNA replication is not completely clear but one function is to promote replication restart [5]. While wtp53 is unstable in normally proliferating cells, following replication stress and DNA damage the wtp53 protein is stabilized and associates with components of the replisome machinery [4]. The wtp53 protein participates in surveillance of replicating DNA and interacts with replication protein A (RPA) to block homologous recombination [6, 7]. Additionally, wtp53 activity results in increased excision of mismatched nucleotides in damaged DNA thus improving replication fidelity [8, 9]. Interestingly, while purified wtp53 is able to block SV40 replication *in vitro,* purified tumor-derived missense mutant p53 (mtp53) proteins do not block SV40 origin of replication (ori) DNA replication or SV40 T-antigen mediated DNA unwinding [10]. While tumor-derived missense mtp53 proteins have altered functions they contain the two N-terminal transactivation domains, followed by a proline rich domain, an altered central DNA binding domain, and the oligomerization domain (OD) and the C-terminal regulatory domain (CTD) [11].

The p53 gene is mutated in 70% of all human cancers [12]. The hot-spot mutations inhibit p53’s sequence-specific DNA binding ability [13]. They also inhibit activation of transcription target genes associated with the initiation of growth arrest and apoptosis [11, 14, 15]. In contrast to the unstable wtp53 protein, the forms of mtp53 found in cancers are also highly stable and often have newfound gained functions [11, 16]. One such gain-of-function (GOF) hot-spot mtp53 is R273H which is often found in triple negative breast cancers [17]. GOF mtp53 R273H promotes oncogenesis partly by interacting with partners of the replication machinery, like PARP, PCNA and MCM2-7 [11, 18–21]. While many studies have examined GOF mtp53 from the perspective of new found transcriptional properties, few have examined how mtp53 directly influences DNA replication and cell cycle progression [11]. Herein we further examine the ability of mtp53 R273H, and its OD and CTD regions, to influence cell proliferation, DNA replication, and cell cycle progression of breast cancer cells.

The C-terminal regulatory domain of wtp53 allows the protein to interact non-specifically with DNA [22–24]. Accordingly, it stands to reason that the subset of mtp53 proteins found in breast cancers would also maintain this non-specific interaction with DNA. Indeed, such mtp53 proteins interact tightly with breast cancer chromatin [18–20, 25]. To date, the domains of the mtp53 protein required for transcription-independent chromatin interactions have not been systematically evaluated. The impetus for these studies was to assess the requirement for mtp53 oligomerization (OD) and C-terminal regulatory (CTD) domains for the function of the mtp53-MCM-PARP1 axis, a putative GOF pathway uncovered and characterized in the mtp53R273H expressing cell lines MDA-MB-468, HT-29, and PANC-1 [18–20]. The choice to investigate a potential role for the OD and CTD domains within the context of the mtp53 R273H allele was two-fold: (1) we delineated the above GOF pathway in this background and in parallel with the studies reported, worked to generate more tools to elucidate the role of each domain in mtp53 GOF activity; (2) our pursuit of a genetic approach using CRISPR-Cas9 technology to create specific alterations within each domain necessitated that we focus first on one mtp53 R273H expressing-cell line (given our limited resources).

We asked if the level and/or the CTD or OD of mtp53 R273H regulates the GOF properties in the triple negative breast cancer cell line MDA-MB-468. MDA-MB-468 cells contain only single mutated copies of mtp53 R273H and no wtp53 allele [26]. CRISPR-Cas9 sgRNA editing of the C-terminal regions of the endogenous mtp53 gene were carried out so as to delete gene sequences that correspond to the OD and CTD regions. In this research perspective we used CRISPR-Cas9 targeting of these C-terminal regions of the mtp53 gene in MDA-MB-468 cells to further extend our findings with shRNA-mediated reduction of mtp53 proteins [18–20]. We saw that a frameshift mutation in C-terminal end of mtp53 reduced stable mtp53 R273H protein levels compared to the parental MDA-MB-468 cells, reduced cell proliferation, and reduced the chromatin association of replication proteins that mirrored their slow progression through S-phase. The CRISPR-Cas9 targeting also produced cell clones with C-terminal truncated mtp53 R273H proteins; such cells with truncated mtp53 R273H showed decreased proliferation as compared to the parental cells but progressed through S phase in a similar manner. It is clear that increased studies are needed to fully uncover the roles played by the multiple domains of mtp53 in GOF oncogenic properties throughout the different cell cycle stages.

## RESULTS

### Cas9-sgRNA targeted alteration of the C-terminal oligomerization (OD) and C-terminal regulatory domain (CTD) of mtp53 R273H within TNBC MDA-MB-468 cells results in novel frame-shift and deletion variant expressing cell lines

**Figure 1:**
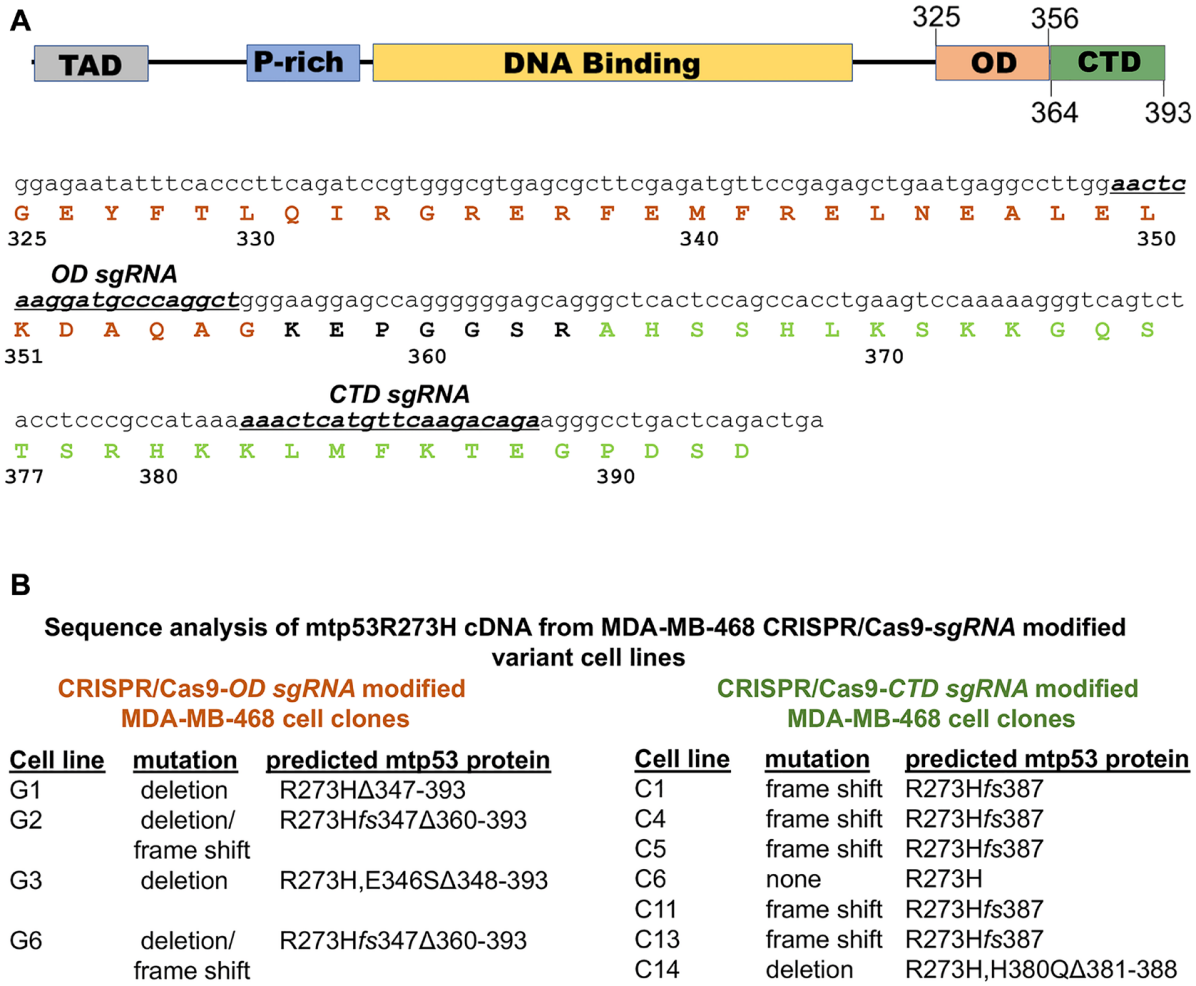
Domain architecture of p53 and nomenclature of clones generated via CRISPR-Cas9. (**A**) Highlights the domain architecture of p53 with emphasis on the sgRNA designed to target the oligomerization (OD) and C-terminal domain (CTD) of mtp53. (**B**) CRISPR-Cas9 was used to generate clones with either OD or CTD mutations. Clones were selected with FACS sorting of eGFP positive cells. Selected clones were named based on the region and type of mutation that resulted.

Previous studies have investigated the dependence of cancer cells on sequence-specific DNA binding-deficient mutant p53 proteins (mtp53) using mtp53-depleted cell lines created with shRNA-based technologies. Although highly informative in revealing gain-of-function (GOF) activities for mtp53 in several metabolic pathways, the capacity of such RNAi-based strategies to yield information about protein functional domains is limited. Using the triple negative breast cancer cell line MDA-MB-468, we employed sgRNA-CRISPR Cas9 technology to generate mtp53 R273H specific alterations in the oligomerization domain (OD) and C-terminal regulatory domain (CTD) (Figure 1A). We targeted within the OD domain amino acid residues 349–355 that mediate wild-type p53 dimer-dimer interactions, and amino acid residues 382–388 within the CTD domain, which contains six conserved lysine residues shown to be required for wild type p53 non-specific DNA binding activity and transcriptional activation of a subset of target genes [27–29]. Following introduction of each sgRNA-Cas9 complex into MDA-MB-468 cells, clone selection and amplification, cDNA from each clone was generated to analyze the resultant OD and CTD sgRNA-directed targeting events. Sequence analysis revealed both deletion and frameshift events with distinct expression characteristics (Figure 1B). For those clones derived from targeting the OD domain (cell lines G1, G2, G3 and G6), we observed a deletion of the entire portion of the gene downstream of the predicted PAM that encodes amino acid residue 347 in three (G1, G3 and G6) and in one a frameshift with a subsequent deletion (G2). Of the clones resulting from CTD sgRNA targeting (cell lines C1, C4, C5, C11, C13, and C14), one was a small deletion (C14) that contained a mutation at residue 380 (H→Q) and a deletion of residues 381–388 and the rest contained frameshift mutations that changed the amino acid residues 387–393 C-terminal to the targeted lysine residues (Supplementary Figure 1).

**Figure 2:**
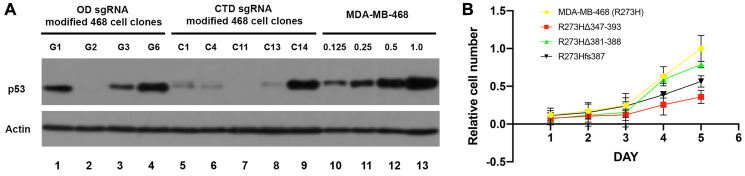
Reduction of mtp53 protein levels and proliferation results from mutations in the OD and CTD of p53. (**A**) Cells were harvested and lysed in RIPA buffer. The protein levels of mtp53 were assessed with western blot analyses. Actin was used as a loading control to normalize the protein being measured. (**B**) The parental R273H, mtp53 R273HΔ347-393, mtp53 R273HΔ381-388 and mtp53 R273H*fs*387 cells were seeded at 1 × 10^5^ cells in 5 ml media on a 6 cm plate and grown for 5 consecutive days. Three independent biological replicates of the cell proliferation assay were performed. Total cell count was done using a hemocytometer.

We examined the size and abundance of predicted mtp53 variants relative to the parental mtp53 R273H MDA-MB-468 cell line by comparing total cell lysates by western blot analysis (Figure 2A). The deletion clones derived from OD domain-sgRNA targeting events were found to produce mtp53 protein with a molecular weight that was consistent with the predicted 47 amino acid deletion (Figure 2A, lanes 1, 3, and 4). In the case of the CTD domain-targeted clones, those containing frameshift mutations (cell lines C1, C4, C5 (not shown), C11, and C13) produced a protein comparable in size to the mtp53 in the MDA-MB-468 parental cells but at a level significantly lower than that of the parental cell line (Figure 2A, lanes 5–8). The most severe reduction in protein level was reproducibly detected for cell lines G2 and C11 (Figure 2A, lanes 2 and 7). The cell line with the small C-terminal in-frame deletion (C14) produced a mtp53 variant protein at a level ~50% of that made by the parental and had a size consistent with its predicted molecular weight (Figure 2A, lane 9). In summary, the CRISPR-Cas9 mediated targeting of mtp53 R273H in MDA-MB-468 cells produced three classes of mtp53 variant expressing cell lines: (1) a large deletion variant R273HΔ347-393 that eliminated part of the OD and completely eliminated the CTD region (cell lines G1, G3 and G6 referred to collectively here as mtp53 R273HΔ347-393 cells); (2) a small deletion variant R273HΔ381-388 that eliminated the last three conserved lysine residues in the CTD domain (cell line C14 referred to here as mtp53 R273HΔ381-388 cells), and (3) a frameshift variant R273H*fs*387 that expresses extremely low levels of mtp53 (cell lines C1, C4, C5, C11, and C13 referred to here as mtp53 R273H*fs*387 cells). We chose for more extensive characterization CRISPR cell lines G1, C14 and C11 as representatives of their respective classes, and examined other clones within each class of cell lines to confirm observed phenotypes.

### CRISPR-Cas9 alteration of the C-terminal OD and CTD domains within mtp53 R273H impairs MDA-MB-468 cell proliferation and mtp53 chromatin binding activity

Previous studies using RNAi-based strategies have shown that depletion or knockdown (KD) from established cancer cell lines expressing mtp53 proteins can impair cell proliferation [18–20, 25]. We therefore compared growth kinetics of the parental mtp53 R273H MDA-MB-468 cells to the three CRISPR cell line classes- mtp53 R273HΔ347-393 (C-terminal OD and CTD deletion), mtp53 R273HΔ381-388 (CTD deletion), and mtp53-depleted R273H*fs*387 (Figure 2B). The mtp53-depleted R273H*fs*387 and mtp53 R273HΔ347-393 cells proliferated significantly slower than the MDA-MB-468 parental cell line (Figure 2B). Thus, these data suggest the CRISPR cell line with barely detectable mtp53 and the one missing a large portion of the protein possess phenotypes shared with RNAi-mediated mtp53 KD cells documented previously by our lab and others [18, 25].

**Figure 3:**
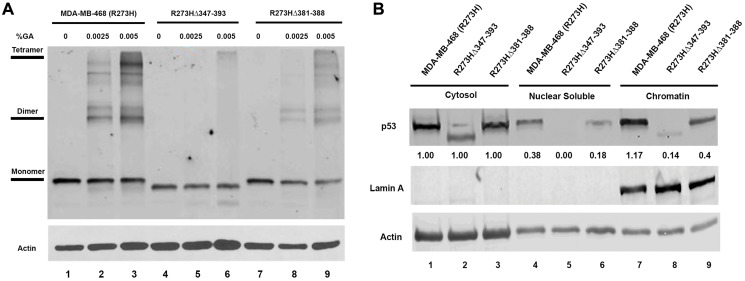
Mutations in the OD of mtp53 destabilize tetramer formation and reduce mtp53 chromatin association. (**A**) The glutaraldehyde crosslinking and western blot analysis were used to determine the oligomerization state of mtp53 in cells either expressing R273H mtp53 or R273H-dual mutants mtp53 R273HΔ347-393 or mtp53 R273HΔ381-388. Glutaraldehyde was added to the lysate at concentrations of 0.00, 0.0025 and 0.005%. (**B**) Chromatin fractionation was used to separate the lysate into the cytosolic, nuclear soluble and chromatin fractions. The mtp53 distribution in the various fractions was assessed with western blot analyses. ImageJ was used to quantify the signal intensities of each band to determine the extent to which the R273H-dual mutant chromatin association varied from the R273H parental. The results are presentative of three independent biological replicates.

It is well documented that both the OD and CTD domains of the wtp53 protein are required for chromatin localization and sequence specific DNA binding activity [29, 30]. However, the relative contribution of either domain to the reported GOF mtp53 activities remains poorly understood. Therefore, we examined the ability of the mtp53 protein variants expressed within the mtp53 R273HΔ347-393 and mtp53 R273HΔ381-388 cell lines to form oligomers and to interact stably with chromatin. The wtp53 protein can be observed as a tetramer through chemical crosslinking methods [31]. The wtp53 protein forms a dimer of dimers [30]. These same chemical crosslinking methods demonstrated that changes to the oligomerization domain of wtp53 results in variants that form either predominantly monomers or dimers [32, 33]. Tetramer formation is essential for many of the wtp53 tumor suppressor functions and therefore it is possible that tetramerization is important for mtp53 GOF [30]. We tested the ability of the GOF mtp53 R273H variants to form tetramers by increasing the concentration of chemical crosslinking agent in lysates from MDA-MB-468 cells. We observed clear formation of mtp53 R273H dimers and tetramers (Figure 3A, lane 1–3). Importantly, we observed that even with increasing chemical crosslinking agent, the mtp53 R273HΔ347-393 MDA-MB-468 CRISPR cells produced a truncated variant that remained as a monomer (Figure 3A, lanes 4–6). On the other hand, the mtp53 R273HΔ381-388 cells produced a mtp53 protein capable of forming both dimers and tetramers, albeit with a lower ratio of tetramers to dimers than mtp53 R273H (Figure 3A, lanes 7–9). We previously used chromatin fractionation to show that mtp53 R273H is tightly tethered to chromatin. We used this same method to determine if tetramerization influenced the interaction of mtp53 with chromatin. We compared the sub-cellular mtp53 distribution in MDA-MB-468 cells with mtp53 R273H to mtp53 R273HΔ347-393 and R273HΔ381-388 cells (Figure 3B). There was substantial full-length and truncated mtp53 present in the cytosol of all three cell lines (Figure 3B, lanes 1–3). However, the monomeric mtp53 R273HΔ347-393 protein was barely detectable in both the nuclear soluble or chromatin bound fractionation (Figure 3B, lanes 5 and 8). When compared to mtp53R273H, the smaller deletion variant mtp53 R273HΔ381-388 showed a less severe reduction in the nuclear soluble and chromatin bound fractionation (Figure 3B, compare lane 4 to 6 and lane 7 to 9).

### Thymidine sensitivity manifested by CRISPR-Cas9 generated MDA-MB-468 mtp53 R273H variant cell lines reveals a requirement for mtp53 for proper S-phase progression

**Figure 4:**
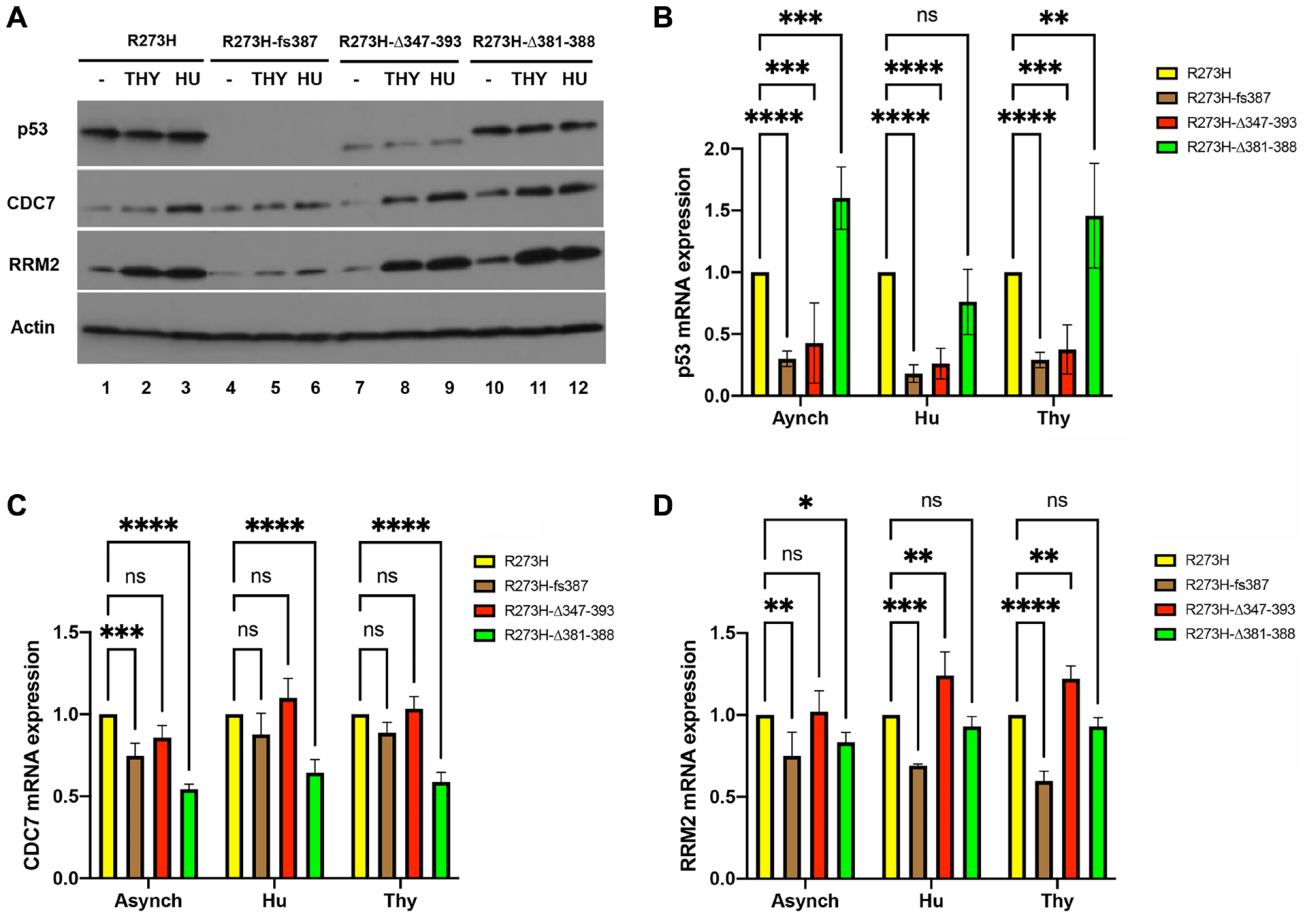
Correlation of mtp53 variants protein and mRNA abundance with RRM2 and CDC7 in MDA-MB-468 CRISPR-Cas9 generated mtp53 cell lines. (**A**–**D**) The relative protein and mRNA abundance of *TP53*, *RRM2* and *CDC7* was examined within asynchronous (lanes labeled (–)) and G1/S synchronized cell populations of parental MDA-MB-468 mtp53 R273H and CRISPR-generated mtp53 R273H*fs*387, mtp53 R273HΔ347-393, and mtp53 R273HΔ381-388 by western blot analysis of total cell lysates (A), and quantitative RT-PCR analysis of mRNA B–D) prepared from each cell population. (B–D) Represent data for three independent biological replicates. A two-way anova with Dunnett’s multiple comparison was performed and the level of significance set at ^*^ *P* ≤ 0.05; ^**^ *P* ≤ 0.01; ^****^ *P* ≤ 0.0001; ns, not significant. Sub-confluent cultures (~ 50% confluent) of each cell line were synchronized at G1/S by treatment of cell populations with either 2 mM thymidine (Thy) or 2 mM hydroxyurea (HU) for 24 hours, harvested and then processed for flow cytometry (Supplementary Figure 2) and the aforementioned analyses above as described in "Materials and Methods".

A number of different activities contribute to mtp53 GOF oncogenic properties including interactions with other transcription factors and/or their regulators, resulting in activation of non-p53 response element target genes [34], as well as interactions with DNA replication and repair proteins like the MCM2-7 helicase and the PARP1 DNA replication stress sensor [35]. In transcription regulation mtp53 acts as a component of transcriptional co-activator complexes to upregulate expression of the gene for the *CDC7* and several genes involved in nucleotide metabolism including the ribonucleotide reductase (RNR) regulatory subunit *RRM2* [36–38]. To assess if the CRISPR cell lines display the transcriptional phenotypes reported for mtp53 RNAi-mediated knockdown cell lines, we compared CDC7 and RRM2 protein and mRNA levels within asynchronous or G1/S synchronized populations of the parental mtp53 R273H and in the CRISPR mtp53 variant expressing cell lines (Figure 4A–4D). Cell populations were treated with thymidine (Thy) or hydroxyurea (HU) to block cells at G1/S (Figure 4 and Supplementary Figure 2). The mtp53 protein levels were unchanged (Figure 4A). The mRNA levels for *TP53* were reduced in the CRISPR mtp53 R273H*fs*387 and the CRISPR mtp53 R273HΔ347-393 which corresponded to the levels of p53 protein expression (compare Figure 4A and 4B). The cell cycle distribution within asynchronous populations was shown to be comparable as determined by flow cytometry (Supplementary Figure 2). As expected, a significant increase in both the protein and mRNA levels of CDC7 and RRM2 was observed in cell extracts from the parental mtp53 R273H G1/S-synchronized cells compared to asynchronous cell populations; mtp53 protein and mRNA levels were unchanged (Figure 4A, lane 1–3 and data not shown). In contrast, the CRISPR mtp53 R273H*fs*387 mtp53-depleted cell line showed a significant reduction in the levels of CDC7 and RRM2 protein (Figure 4A, lanes 4–6). When compared to parental R273H cells the CRISPR mtp53 R273H*fs*387 cells also displayed a statistically significant reduction of *CDC7* mRNA in asynchronous cells and a decrease in *RRM2* mRNA in all cell cycle conditions tested (Figures 4C and 4D). The CRISPR mtp53 R273HΔ347-393 and mtp53 R273HΔ381-388 displayed G1/S-dependent expression of CDC7 and RRM2 protein at levels comparable to that of the parental R273H cells (Figure 4A, lanes 7–12). The CRISPR mtp53 R273HΔ347-393 also showed *CDC7* mRNA levels comparable to that of the parental R273H cells (Figure 4C) but showed a moderate increase in *RRM2* mRNA in Thy and HU synchronous cells (Figure 4D). Therefore, the decreased proliferation of CRISPR mtp53 R273HΔ347-393 could not be correlated to reduced CDC7 or RRM2 levels. However, the CRISPR mtp53 R273HΔ381-388 cell line showed a statistically significant decrease in *CDC7* mRNA in all the conditions tested and also in *RRM2* mRNA in asynchronous cells (Figures 4C and 4D).

**Figure 5:**
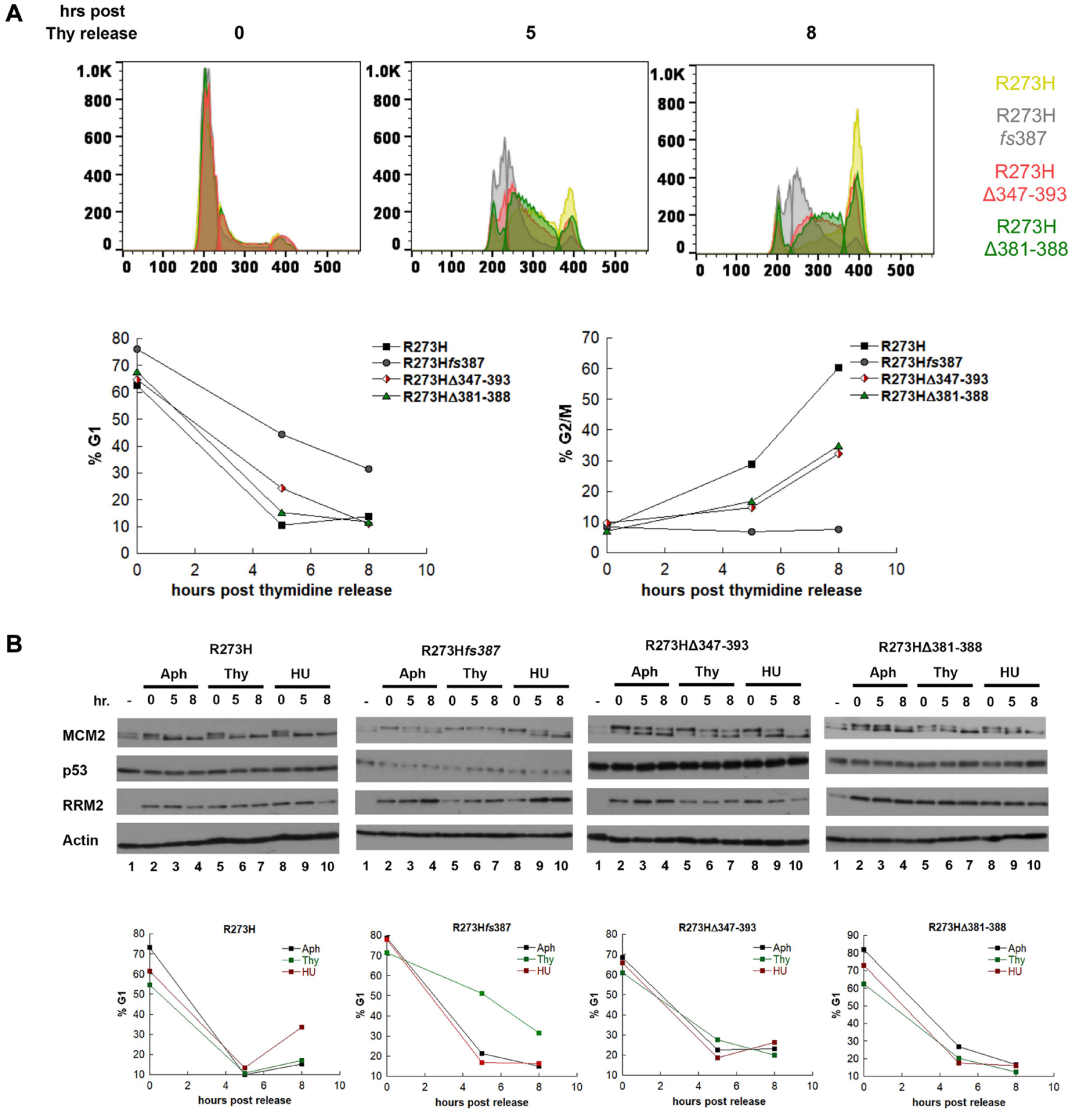
MDA-MB-468 CRISPR-Cas9 generated mtp53 variant-expressing cells display thymidine sensitivity characterized by slow progression through S-phase. (**A**) The kinetics of S-phase progression of parental and CRISPR variant cell lines parental MDA-MB-468 mtp53 R273H and CRISPR-generated mtp53 R273H*fs*387, mtp53 R273HΔ347-393, and mtp53 R273HΔ381-388 were compared post synchronization of 50% confluent cultures of each with 2 mM thymidine. At time points 0, 5, and 8 hours post release from the Thy block, cell populations from each cell line were harvested simultaneously and the cell cycle distribution of PI-stained cells was determined by flow cytometry. The distribution of cells within the G1, S, and G2/M phases at each time point is represented in the super-imposed histograms of mtp53 R273H cells (yellow), mtp53-depleted cells R273H*fs*387 (gray), mtp53 R273HΔ347-393 cells (red), and mtp53 R273HΔ381-388 (green), with the percentage of G1 and G2/M cells at each time points for each cell line presented in the graphs on the right. (**B**) The abundance of the p53, MCM2 and RRM2 proteins were examined in extracts from asynchronous (–) and G1/S synchronized cell populations harvested 0, 5, and 8 hours post release from a 24 incubation with the cell cycle inhibitors aphidicolin (Aph), Thymidine (Thy), or hydroxyurea (HU). Parental MDA-MB-468 mtp53 R273H and CRISPR-generated mtp53 variants cell lines mtp53-depleted R273H*fs*387 cells, mtp53 R273HΔ347-393 cells, and mtp53 R273HΔ381-388 cells, were cultured to 50% confluency before addition of either 5 μM Aph, 2 mM Thy, or 2 mM HU, and at the above time points cell populations were harvested and processed for either cell cycle analysis by flow cytometry, or western blotting. The distribution of cells within G1, S, and G2 based on propidium iodine (PI) staining was determined as described in "Materials and Methods", and the percentage within G1 and G2 for each time point is represented. All extracts were analyzed by SDS-PAGE on 10% gels, and subject to western blot analysis. The experiment represented in (A) were done twice for all cell lines and in (B) they were performed twice for the CRISPR mtp53 R273H*fs*387 variant and one time for all others.

Because of the observed decrease in both *RRM2* mRNA and protein observed for CRISPR mtp53 R273H*fs*387, we asked if RRM2 activity is limiting for their S-phase progression by synchronizing the parental R273H and CRISPR mtp53 variant cell lines with thymidine and using flow cytometry to monitor their S-phase progression post inhibitor removal (Figure 5A). We did this in order to make a direct comparison of the kinetics of S-phase progression of the CRISPR mtp53 variant cell lines to the parental post thymidine synchronization. Direct comparison of the kinetics of S-phase progression of the CRISPR mtp53 variant cell lines to the parental post thymidine synchronization uncovered that all three CRISPR cell line classes are thymidine sensitive, with the CRISPR mtp53-depleted R273H*fs*387 displaying the highest thymidine sensitivity phenotype and the mtp53 R273HΔ381-388 showing the least sensitive phenotype (Figure 5A; histograms for all individual cell lines present in Supplementary Figure 3). Figure 5A shows the outcomes at times 0, 5 and 8 hours post inhibitor removal, and the histograms are presented as overlapping diagrams. At time 0 the cell cycle profiles of the four cell lines overlap, however, by 5-hours and 8-hours post release, a shift is visible demonstrating that all three CRISPR cell line classes showed varying degrees of thymidine sensitivity and slower progression through S-phase (Figure 5A and Supplementary Figures 3 and 4). We observed that the parental MDA-MB-468 cells progressed the most rapidly (Figure 5A, see yellow peak and graphs showing R273H fast reduction in % G1 and fast increase in %G2 cell populations). We observed that the mtp53-depleted R273H*fs*387 cells (with low RRM2 protein and mRNA) progressed the most slowly (Figure 5A, see grey peak and graphs showing R273H*fs*387 cells with slow reduction in % G1 and slow increase in %G2 cell populations). The mtp53 R273HΔ347-393 and R273HΔ381-388 cells demonstrated an intermediary phenotype for their cell cycle progression following the thymidine release (Figure 5A).

We further asked if RRM2 activity is limiting for S-phase progression by synchronizing with aphidicolin in addition to Thy and HU. We also used aphidicolin (Aph, a polymerase α/primase inhibitor) for cell synchronization at G1/S to assess the dependence of our observations on usage of RNR inhibitors. The synchronized cells were then monitored for their S-phase progression post inhibitor removal by flow cytometry and western blot analysis of whole cell lysates for p53, RRM2 and MCM2 (Figure 5B). First, despite the lower abundance of RRM2 within CRISPR mtp53 R273H*fs*387 cells, we observed that the level of RRM2 was not limiting for S-phase progression, because when synchronized with aphidicolin, parental mtp53 R273H and CRISPR mtp53 R273H*fs*387 cells were observed to proceed through S-phase with similar kinetics (Supplementary Figure 5 and Figure 5B). In addition, although CRISPR mtp53 R273H*fs*387 cells had lower RRM2 levels in Thy synchronized cells, an increase in the abundance of RRM2 protein was observed as Aph- and HU-synchronized CRISPR mtp53 R273H*fs*387 cells as they proceeded through S-phase (Figure 5B). This demonstrated that RRM2 expression during S-phase is independent of mtp53 R273H expression level. We found RRM2 protein abundance in CRISPR mtp53 R273H*fs*387 cells correlated with slow S-phase progression in response specifically to thymidine synchronization (a phenotype that we refer to as thymidine sensitivity). As shown in Figure 5B, mtp53 R273H*fs*387 cells demonstrated a significantly higher percentage of cells with a G1 DNA content (≥ 50%) 5 hours post release from the thymidine block compared to that (~20% G1 DNA content cells) at the same time point post release from Aph and HU.

We also asked if the MCM2 replication helicase activation, that we previously documented reduced in shRNA KD GOF mtp53 R273H cells, was also reduced in the CRISPR mtp53 R273H*fs*387 cells. Flow cytometry and western blot analysis of whole cell lysates for the proteins indicated that the MCM2 helicase was functioning differently in parental R273H cells and CRISPR mtp53 R273H*fs*387 cells (Figure 5B). As one of the primary targets of cell cycle regulation of DNA replication, the MCM2 protein has been shown to be subject to multiple phosphorylation events by the CDC7/Dbf4 kinase, whose function in DNA replication is to commit cells to initiate DNA synthesis at licensed origins by activating the MCM complex [39, 40]. The hyper-phosphorylated form of MCM2 migrates paradoxically faster on SDS-PAGE gels and its measurement can be used as an additional indicator of the extent of S-phase progression [39]. The hyper-phosphorylated form of MCM2 was clearly evident in parental MDA-MB-468 R273H cell whole cell lysates and complete conversion occurred by 8-hours post Aph, Thy, or HU (Figure 5B, R273H lanes 4, 7, and 10). Moreover, FACS analysis demonstrated that by 5-hours post Aph, Thy, or HU removal all the cells had proceeded out of the G1 phase of the cell cycle (Figure 5B, graph below R273H western blot). Strikingly, in CRISPR mtp53-depleted R273H*fs*387 cells the hyper-phosphorylated form of MCM2 was not evident in the 8-hours post Thy (Figure 5B, whole cell lysates R273H*fs*387 compare lane7 to lanes 4 and 10). The R273H*fs*387 Thy block 8-hours post-release revealed virtually all MCM2 protein migrating with the slower unphosphorylated form, suggesting less origin firing and consequently slower S-phase progression. Moreover, FACS analysis demonstrated that by 5-hours post Thy removal less than 50% of the cells had proceeded out of the G1 phase of the cell cycle (Figure 5B, graph below R273H*fs*387 western blot). Apparent slower S-phase progression was also manifested by other CRISPR mtp53-depleted cell lines (Supplementary Figure 4), and by the CRISPR mtp53 variant cell lines, albeit to a lesser extent (Figure 5). The MCM2 in mtp53 R273HΔ347-393 was incompletely converted to the hyper-phosphorylated form by 8-hours post release from Aph, Thy, or HU (Figure 5B, mtp53 R273HΔ347-393 lanes 4, 7, and 10 see appearance of both MCM2 isoforms). Additionally, FACS analysis demonstrated that by 5-hours post agent removal there was incomplete progression out of the G1 phase of the cell cycle (Figure 5B, graph below mtp53 R273HΔ347-393 western blot). The R273HΔ381-388 small C-terminal deletion mutant had a cell cycle progression profile most similar to the MDA-MB-468 R273 parental cells (Figure 5B, R273HΔ381-388). Taken together, by building an S-phase profile through usage of more than one cell synchronization method, and multiple CRISPR clones, our data suggest a role for mtp53 R273H levels in an early step in S-phase while potentially also being regulating at other cell cycle stages by amino acids in the mtp53 OD and CTD regions.

### Impaired replication factor chromatin assembly underscores the slow S-phase progression of CRISPR-Cas9 generated MDA-MB-468 mtp53 R273H depleted cells

**Figure 6:**
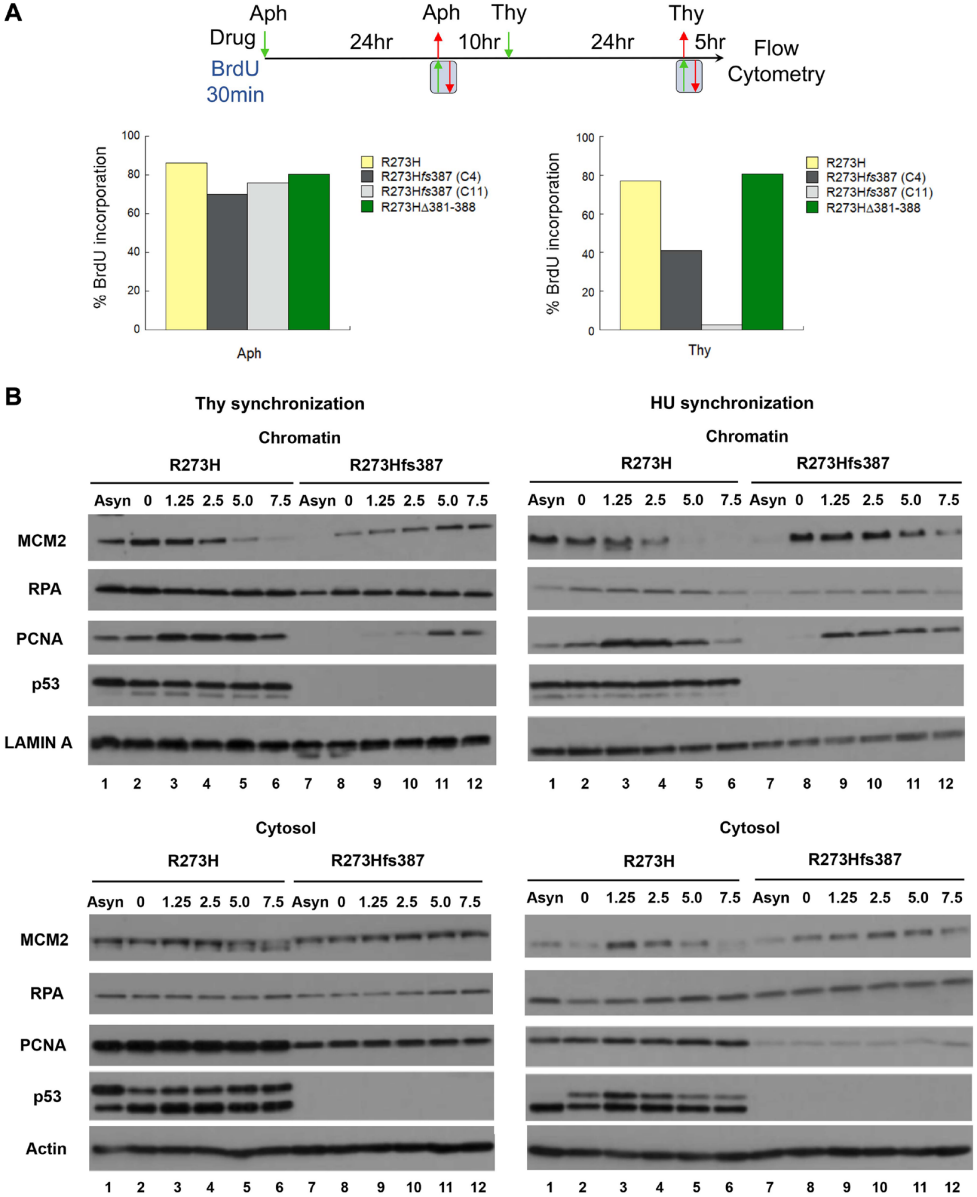
Defective DNA replication underscores the thymidine sensitivity of MDA-MB-468 CRISPR-Cas9 generated mtp53-depleted cells. (**A**) A direct assessment of the S-phase population was determined using flow cytometry by measuring the percentage of parental MDA-MB-468 mtp53 R273H and CRISPR-generated mtp53 variant mtp53-depleted C4 and C11 R273H*fs*387, and mtp53 R273HΔ381-388 cells that incorporate BrdU 5 hours post release from a double Aph-Thy block. After the first synchronization at G1/S with aphidicolin, all cell populations were released into the cell cycle for 10 hours (a period sufficient for bulk genome duplication) before initiation of the second block with thymidine. Following release from either the first or second block, cells were pulse-labeled with BrdU for 30 min and then harvested either immediately thereafter in the case of samples released from a single Aph-block, or after 5 hours in the case of double-block samples labeled post-release from the Thy-block. Histograms represent the percentage of cells within each population that incorporated BrdU. (**B**) Assembly of DNA replication factors onto chromosomes of parental MDA-MB-468 mtp53 R273H and CRISPR-generated mtp53 mtp53-depleted R273H*fs*387 cells during S-phase was measured using the chromatin fractionation assay. Cytosolic and chromatin fractions were prepared as described in "Materials and Methods" from mtp53 R273H and mtp53-depleted R273H*fs*387 cell populations proliferating asynchronously (samples in lanes represent by (–)) or harvested 0, 1.25, 2.5, 5, and 7.5 hours post release from either HU- or Thy- G1/S synchronization, and then analyzed by SDS-PAGE and western blotting for the indicated proteins. The experiment in (A) was performed twice and that in (B) for two biological replicates with Thy-synchronized cells. Chromatin fractionation often resulted in multiple different migration forms of p53 protein. We think that this occurs due to do with both alternatively posttranslational modified isoforms and degradation products of p53 (but have not verified the reasons). The chromatin bound p53 showed less variation than the cytosolic p53.

Given that slow S-phase progression defines the thymidine sensitivity of the CRISPR mtp53 R273H*fs*387 variant cell line, we compared R273H*fs*387 to the parental R273H cells for defects in DNA replication using two approaches (Figure 6): (1) flow cytometry to measure the incorporation of the nucleotide precursor bromodeoxyuridine (BrdU) (Figure 6A); and (2) chromatin fractionation to measure the assembly of the MCM helicase, RPA (single-strand DNA binding protein), and the PCNA (DNA polymerase processivity clamp) onto chromatin during S-phase (Figure 6B). To maximize enrichment of cells at G1/S, we used a double Aph-Thy block synchronization protocol in which mtp53 R273H, and CRISPR mtp53 R273H*fs*387 (C4 and C11 cell lines) and R273HΔ381-388 cells were first blocked with aphidicolin and then released into S-phase for a period sufficient for completion of DNA replication before initiation of the second block with thymidine. Following release from the second block, cells were incubated with BrdU for the first 30 min of S-phase (BrdU labelling), and after removal, allowed to continue in S-phase for an additional 4.5 hours. Comparable synchrony of all cell populations examined was assessed and confirmed by BrdU labelling after the first block, and then measuring BrdU incorporation after completion of the second block (data not shown). All the cell lines examined showed indistinguishable levels of BrdU incorporation when parallel cultures of each were labelled following the first block with Aph (Figure 6A, Aph). Consistent with a defect in DNA replication following the second block with thymidine, the CRISPR mtp53-depleted R273H*fs*387 cell lines displayed poor BrdU incorporation (41.1% cell line C4 which expressed more mtp53 and 2.6% cell line C11 which expressed less mtp53 as seen in Figure 2A), compared to the parental mtp53 R273H (77.1%) and the CRISPR R273HΔ381-388 cells (80.6%) (Figure 6A, Thy).

We next compared the kinetics of assembly of DNA replication factors on the chromatin for R273H expressing and mtp53-depleted R273H*fs*387 cells (Figure 6B). The temporally ordered assembly and disassembly of replication proteins provides a molecular profile that informs defects in DNA replication [40–42]. Following Thy or HU synchronization of CRISPR mtp53 R273H*fs*387 and parental mtp53 R273H cells, we prepared cytosolic and chromatin fractions at the indicated time points post release, and performed for each western blot analysis for RPA, the MCM2 subunit of the MCM helicase, and the PCNA. Assembly of the MCM complex onto chromatin isolated from HU synchronized R273H*fs*387 and parental cells manifested similar kinetics, with the peak of MCM2 on chromatin found before block release at the onset of S-phase, and persisted on chromatin up to 2.5 hours post release (Figure 6B, right panel compare chromatin lanes 1–6 to 7–12). Dissociation of the complex, indicative of completion of DNA synthesis within replicons, commenced after 2.5 hours, and correlated with the hyperphosphorylated form of MCM2 in the cytosol. Likewise, the kinetics of PCNA association with chromatin, which temporally follows MCM2, was observed within R273H*fs*387 cells to mirror that of the parental cells when synchronized with HU, with peak loading detectable 1.25–2.5 hours post release (Figure 6B, right panel compare lanes 1–6 to 7–12). Surprisingly however, a stark change in the kinetics in replication protein chromatin assembly was found following thymidine synchronization (Figure 6B, left panel compare chromatin lanes 1–6 to 7–12. Unlike in the parental mtp53 R273H cells in which the temporally ordered assembly and disassembly was kinetically similar to that displayed by HU synchronized cells, a measurable delay in the assembly of both MCM2 and consequently PCNA on chromatin was observed in the CRISPR R273H*fs*387 cells, with significant loading of each occurring only 5 hours post release without detection of a discernable peak (Figure 6B, compare chromatin lanes 1–6 to 7–12). In the released thymidine synchronization samples of mtp53-depleted R273H*fs*387 cells, the MCM2 subunit of the MCM helicase and PCNA showed reduced chromatin loading. In cell fractionated parental R273H samples the phosphorylated MCM2 ended up more prominent in the cytosolic fraction, which agrees with past evidence that phosphorylated MCM2 isoforms are not stably associated with chromatin [39] (Figure 6B, compare cytosol lanes 1–6 to 7–12). The mtp53-depleted R273H*fs*387 cells had a clear reduction in cytosolic phosphorylated MCM2 and a measurable delay in the assembly of both MCM2 and PCNA on chromatin. In the parental mtp53 R273H cells significant loading of MCM2 and PCNA occurred immediately. On the other hand, in mtp53-depleted R273H*fs*387 cells it took 5 hours post Thy release for PCNA to load. For the Thy released parental mtp53 R273H cells completion of DNA synthesis within replicons occurred by 2.5 hours but for the mtp53-depleted R273H*fs*387 cells the completion of replication was significantly delayed. Taken together, our analysis suggests that in response to thymidine exposure, mtp53 promotes DNA replication by stimulating replisome assembly and therefore mtp53 R273H is required for S-phase progression under specific types of replication conditions (that remained to be defined).

## DISCUSSION

We recently discovered that mtp53 R273H interacts with replicating DNA and increases the recruitment of replication associated proteins to chromatin [18, 19]. Therefore, contemplating which wtp53 functions are, and are not, maintained in mtp53 R273H may help to explain the replication-associated mtp53 GOF activities. Wtp53 can behave as a survival factor that assists cells in accommodating replication stresses of different forms [2–4, 43, 44]. It is well known that wtp53 functions as a transcription factor but there are also many reports that describe alternative functions of wtp53 in regulating DNA replication and repair [1–9]. The amino terminus of wtp53 interacts with replication-associated proteins PCNA, RPA, as well as DNA Polymerases iota and beta [43]. The carboxyl-terminus of wtp53 interacts with PARP1 [45]. The amino and carboxyl regions of GOF mtp53 are unchanged for most GOF mtp53 proteins (as is the case for GOF mtp53 R273H described in this perspective report) [11]. Herein, we extended our finding that mtp53 R273H interacts with replicating DNA by using CRISPR-Cas9 mutants. We altered the endogenous locus of mtp53 R273H in the triple negative breast cancer cell line MDA-MB-468 by CRISPR-Cas9 targeting of the OD and CTD regions. We examined how changes in the level of mtp53 R273H level and/or deletion of the CTD, or OD plus CTD, region influenced cell proliferation, cell cycle progression, and chromatin association of mtp53, RPA, PCNA and MCM2. Targeting the CTD resulted in the outcome of the frameshift mutation, mtp53 R273H*fs*387, which caused a lower level of mRNA transcript. This was possibly due to mRNA alterations through the non-stop or no-go mRNA decay pathway which detects when elongation is not proceeding properly [46, 47]. We observed reduced mtp53 protein level for mtp53 R273H*fs*387 cells, and reduced proliferation in the associated CRISPR-Cas9 clone (see Figures 1 and 2). Targeting the CTD produced a mtp53 R273HΔ381-388 with a small deletion of the CTD which also reduced cell proliferation. The OD targeting produced a large deletion of the OD plus CTD giving mtp53 R273HΔ347-393 cells which also reduced cell proliferation (see Figures 1 and 2).

The findings thus far for the association of mtp53 with respect to the regulation of DNA replication includes increasing the association of replication proteins with chromatin and improving nucleotide metabolism to generate free nucleotides [18–21, 36, 37]. Herein we see that frameshift mutations that reduced mtp53 R273H to barely detectable levels correlated with decreased MDA-MB-468 cell proliferation and reduced RRM2 protein and mRNA (Figures 2, 5 and 6). Moreover, when such cells were synchronized in late G1 by thymidine and then released into S-phase they experienced delayed replication factor stable chromatin assembly as evidenced by a reduction in MCM2 and subsequent PCNA chromatin association (Figure 6). This correlates with a thymidine-release sensitivity and slower progression through the S-phase that was not observed after either HU or aphidicolin S-phase release. Treatment of cells with the different replication inhibitors thymidine, HU, and aphidicolin allows for the comparison of replication stress initiated in different ways [48]. The exact mechanism underlying the apparent cell proliferation defect inferred from the slow growth phenotype displayed by the MDA-MB-468 mtp53 R273Hfs387 and R273HΔ347-393 cell lines is not experimentally addressed in this manuscript. However, compelling models based on previously reported transcription and non-transcription-dependent mtp53 GOF activities have been described, and conceivably loss of function of more than one may underpin the observed cell growth defect. Moreover, the execution point of such activities could be at any stage of the cell cycle, and therefore unearthing the cell proliferation defect mechanism necessitates a comprehensive cell cycle characterization. Our desire to gain mechanistic insights into the DNA replication role of mtp53 R273H and thus extend our findings reported in Xiao et al. limited our cell cycle analysis to S-phase.

Cell synchronization has traditionally utilized drugs that target DNA replication by: (1) inhibiting priming of DNA synthesis and therefore limiting replicative polymerase function (aphidicolin); or (2) inhibiting RNR activity through active site poisoning (HU-mediated tyrosyl radical inactivation) or substrate-level feedback inhibition (thymidine), both of which reduces dNTP pools [49, 50]. Aphidicolin blocks the DNA polymerase and is known to cause hyper-unwinding of the DNA due to uncoupling of the helicase from the polymerases, resulting in expansion, contraction and under-replication of repetitive regions of the genome [51–53]. Both HU and thymidine can cause nucleotide misincorporation (including incorporation of ribonucleotides) and polymerase slippage in the case of thymidine resulting in small deletions and insertions [49, 50, 54–56]. High levels of thymidine also allow for a sneak through the G1 arrest into S-phase [57]. Unrepaired DNA damage detected in the subsequent cell cycle can activate ATM-dependent cell-cycle checkpoints that provide time for repair of damage by excision repair pathways active in G1 [58, 59]. Thymidine treatment slows down replication and release from this form of stress has a longer recovery time than release from either HU or aphidicolin [48]. This may be because helicases overcome thymidine-induced replication stress and this resolution does not involve the use of double strand breaks and double strand break repair [60]. HU scavenges for the free radicals produced by the ribonucleotide reductase pathway and causes the reduction of all nucleotides and causes DNA damage [48].

The wtp53 protein responds differently to alternative forms of replication stress and this can inform our thinking about the roles of mtp53. For example wtp53 can cause a reversible cell cycle arrest in response to ribonucleotide depletion by inducing transcription of the cyclin dependent kinase inhibitor p21 [61]. Genotoxic damage, on the other hand, can signal to the wtp53 pathway to promote DNA repair, both replication dependent and independent, through multiple DNA-damage response pathways [62]. The wtp53 protein can block initiation of replication of viral genomes, but for mammalian genomes wtp53 can either increase fork processivity or slow down DNA synthesis while promoting DNA damage tolerance [2–5, 10, 43]. The observed differences in the presence of wtp53 may depend on the source of replication stress used in the experimental system. For example, in the presence of DNA adducts introduced by an alkylating agent, wtp53 slows down nascent DNA synthesis and may use the 3′–5′ exonuclease activity to assist in repair and DNA damage tolerance [43, 63]. This is controlled in part by the ability of wtp53 to interact with RPA and PCNA through its N-terminal region [43]. Interestingly, mtp53 R273H does not have the ability to slow down nascent DNA synthesis (even though it has the domains available to interact with both RPA and PCNA) [43]. In cells blocked with HU and released into S-phase, wtp53 allows for increased processivity of replication forks [3]. While in cells that are undergoing metabolic stress by the loss of growth factors, the loss of wtp53 activity reduces the amount of DNA damage that occurs in the presence of replication stress [44]. This allows the cells to continue to grow and form large tumors. It could be that by recruiting replication factors, wtp53 can adjust the response of cells as needed. In contrast, when there are DNA adducts then the lesions may signal wtp53 to slow down the replication speed to give time for recombination [43]. However, since HU release gives rapid replication, wtp53 may help the cells to progress rapidly [3, 48]. In all cases, wtp53 helps to alleviate particular types of replication stress through different mechanisms. Therefore, it is to be expected that different forms of replication stress will also signal to mtp53 and the replication progression outcomes differently (Figures 5 and 6). It may be that what mtp53 and wtp53 have in common is what actually accentuates the mtp53 GOF phenotype. This would stand to reason because the GOF mtp53 proteins are highly stable and present in high levels in cancer cells.

Although wtp53-modulated excision repair pathways (such as BER) can occur within G1, those requiring extensive DNA synthesis such as HR occur primarily in the latter half of S-phase and G2 once a donor template for repair is available [64]. How the repair functions of wtp53 are regulated during the cell cycle remain unclear but cell cycle clearly modulates repair choice [58, 64, 65]. Nonetheless, given that wtp53 interactions with replication proteins does not require sequence-specific DNA binding activity, we predict that repair functions in the context of mtp53 may exist absent cell-cycle regulatory signals contributed by wtp53. This would therefore contribute to mtp53 GOF properties because it would help cells survive by aberrantly repairing DNA as a way to sustain cell proliferation. For instance, it has been reported that mtp53 can subvert normal cell-cycle checkpoints to promote unscheduled origin activation [21]. Similarly, we posit that upon S-phase entry mtp53 may recruit DNA repair machinery components (such as error-free and error-prone polymerases beta and pol iota respectively) to activated origins and promote damage repair and therefore enhance replication fork function. In both cases, by exploiting a vestigial function (which in wtp53 is designed for execution in late S-G2 phase to preserve genome integrity as cells advance towards mitosis) mtp53 uses these activities at S-phase entry to expedite chromosomal replication, presumably at the expense of the quality control mechanisms that coordinate the spatio-temporal regulation of replication with DNA repair pathways [59]. Consistent with this idea, it is interesting that we observed the parental MDA-MB-468 R273H cells progress through S-phase fastest when synchronized with RNR inhibitors, which produce a type of damage repaired by excision repair pathways such as BER, NER, and MMR that are active within G1 (BER, NER) and throughout S-phase (BER and MMR) respectively [58, 66]. Moreover, the slower progression through S-phase demonstrated by the R273HΔ347-393 and R273HΔ381-388 CRISPR cell lines in response to thymidine may suggest a role for the C-terminus of mtp53 in repair and/or bypass of damage monitored by such repair pathways, leaving damage to be dealt with in late S/G2 phase.

Unanticipated from these cell cycle studies was the impaired S-phase entry in response to thymidine exposure displayed by the CRISPR R273H*fs*387 that express very low levels of mtp53. Our acquisition of different results using two different RNR inhibitors for G1/S synchronization reflect their distinct modes of action: whereas HU treatment depletes total dNTP pools, excess thymidine causes an imbalance in the nucleotide pools, both dNTP and NTP. Imbalanced dNTP pools have been shown to be highly mutagenic by their ability to cause escape from the S-phase checkpoint, leading to replicative stress in the subsequent cell cycle if not repaired [52, 67]. Recently, a metabolite of pyrimidine nucleotide pool imbalance, the dihydropyrimidines (both uracil and thymidine), have been shown to cause DNA-protein crosslinks that can cause both DNA replication and transcriptional stress if left unrepaired [68]. Interestingly, cells enter S-phase with such damage despite robust wtp53 and Chk1 activation, cellular responses shared by cells in which the Impaired Ribosome Biogenesis Checkpoint (IRBC) has been activated by transcriptional stress induced by inhibitors of purine or pyrimidine biosynthesis [66]. Activation of the IRBC was found to halt S-phase entry through wtp53 activation of the cyclin dependent kinase inhibitor p21 [66]. However, upon p21 degradation, entry into S-phase is accompanied by robust Chk1 activation and sustained wtp53 stabilization in response to the ensuing replicative stress. We posit that in this context the wtp53 stimulates DNA repair mechanisms in response to the replicative stress. Such a role for wtp53, if it does not require its transactivation function, may also be performed by mtp53 in response to imbalanced nucleotide pools. Thus, the fact that mtp53 is not able to halt S-phase entry and may still promote repair as a GOF activity provides a potential mechanism for the phenotype demonstrated without this action in mtp53-depleted CRISPR R273H*fs*387 cells.

We observed that the mtp53 R273H*fs*387 MDA-MB-468 cells have difficulty adapting in response to thymidine treatment release. One potential explanation for the differential response to thymidine release in comparison to HU and aphidicolin is the presence of multiple fork protection mechanisms that may be influenced by mtp53 R273H expression [69]. This protection may afford better recruitment of MCM and PCNA when there are not mismatched nucleotides as can occur during thymidine treatment. The protection mechanisms driven by mtp53 proteins remain to be elucidated. The different replication inhibitors cause subtle differences in replication stress and perhaps alter DNA replication fork architecture in ways that then result in different chromatin protein recruitments to allow for replication stress tolerance. The fact that we identified a mtp53-PARP-MCM axis on replicating DNA and that PARP is involved in lagging strand replication [70] supports this possibility that helicase and lagging strand stress tolerance may be a target for mtp53 R273H related stress resolution.

The wtp53 DNA damage tolerance pathway that decreases nascent DNA synthesis through 3′ to 5′ exonuclease is not facilitated by mtp53 R273H [63]. The wtp53 DNA damage tolerance pathway requires p53 amino acids in the amino terminus that interact with PCNA, RPA, and DNA Polymerases iota and beta [43]. It is not yet clear what component of this activity might remain in the presence of high levels of mtp53 R273H in the absence of exonuclease activity. We observed that when the OD and CTD regions were deleted the mtp53 R273HΔ347-393 protein was a monomer that no longer interacted with chromatin (Figure 3). Furthermore, monomeric mtp53 R273HΔ347-393 cells proliferated almost as slowly as the mtp53 R273H*fs*387depleted cells (Figure 2). However, they progressed through S phase better than mtp53 R273H*fs*387depleted cells. This suggests they may have problems with mitotic exit. Our data makes it clear that the level and structure of mtp53 are involved in the ability of the cancer cells to proceed through the cell cycle and also to proliferate more rapidly. Our studies examined how mtp53 influences replication at the beginning of S-phase in cells that have been arrested by different mechanisms. The results present us with a new perspective with which to consider what mtp53 does in the context of cell cycle and DNA replication regulation. MDM2 and MDMX are two additional proteins in the p53 pathway that directly regulate DNA replication [71–73]. It remains possible that MDM2 and MDMX work together with mtp53 to allow cancer cells to accommodate increased replication stress. There is also the possibility that GOF mtp53 may improve progression through mitosis, but this is not evaluated herein. Future research is required to evaluate how mtp53 regulates all phases of DNA metabolism during the cell cycle. Such research will help to determine the multiple roles of mtp53 in response to different forms of replication stress. Directly introducing specific changes to the different domain of endogenous mtp53 by CRISPR-Cas9 coupled with homologous directed recombination has the potential to improve our understanding about the multiple roles of different GOF mtp53 proteins in different types of cancers.

Our current studies do not point to a specific function executed by the OD and CTD domains in response to thymidine; however, we can show that their loss does not impact replisome assembly at the onset of S-phase as measured by PCNA chromatin loading and we will address this finding in the future. Thus, OD and CTD domain function(s) correlate with events post S-phase entry, in contrast with that function conferred by other p53 domain(s) deficient in the mtp53fs387 cell line, whose loss impedes S-phase entry. Although currently we are unable to articulate the precise roles of these distinct regions of p53 in response to thymidine, our studies suggest that they may function at temporally distinct stages of S-phase.

## MATERIALS AND METHODS

### CRISPR-Cas9 mutagenesis of mtp53 R273H in MDA-MB-468 cells

In order to create dual mutants of p53 we began with human breast cancer cell line MDA-MB-468 purchased from ATCC (https://www.atcc.org/). Authentication and Mycoplasma testing of cell line were done prior to the CRISPR-Cas9 experiment (Genetica DNA Laboratories). We targeted the oligomerization domain (OD) or C-terminal regulatory domain (CTD) by CRISPR-Cas9 mutagenesis using *in vitro* assembled single guide RNA (sgRNA) plus purified Cas9 enzyme from *Streptococcus pyogene* (NEB), 2000 pmol, M0646M EnGen Spy Cas9 NLS which contains Simian virus 40 (SV40) T antigen nuclear localization sequence (NLS) on the N- and C- termini of the protein for targeted ribonucleoprotein (RNP) particles. The MDA-MB-468 cells were trypsinized, washed in 1X PBS without Ca^2+^ and Mg^2+^ and resuspended at 3 × 10^7^ cells/ml. 1 million cells were used per nucleofection in 100 μl of R buffer for the Thermo Fisher Neon Transfection System. Two different sgRNAs were designed and 20 μl of 100 μM each were mixed with 5 μl of purified Cas9 (20 μM) enzyme buffer and incubated for exactly 10 minutes at room temperature and then placed on ice. For each transfection 10 μl of the RNP mixture and 5μg of eGFP-Puro plasmid were added. The sgRNA for the C-terminal region p53 sequence 5′AAACTCATGTTCAAGACAGA3′ (from AA 382-388) was from IDT as Alt-R CRISPR-Cas9 sgRNA: mA*mA*mA*rCrUrCrArUrGrUrUrCrArArGrArCrArGrArGrUrUrUrUrArGrArGrCrUrArGrArArArUrArGrCrArArGrUrUrArArArArUrArArGrGrCrUrArGrUrCrCrGrUrUrArUrCrArArCrUrUrGrArArArArArGrUrGrGrCrArCrCrGrArGrUrCrGrGrUrGrCmU*mU*mU*rU. The sgRNA to the oligomerization domain p53 sequence 5′AACTCAAGGATGCCCAGGCT3′ (from AA 349–355) was from Synthego using EZ kit modified proprietary technology. The transfections were carried out with Thermo Fisher Neon buffers and following Neon conditions 1700V, 20ms, and 1pulse. The transfected cells were added to 2 ml of prewarmed media with no antibiotics in a six well dish. The following day selection was started with 2 μg/ml of puromycin which remained on the cells for 3 days and then cells were sterile sorted for GFP positives. The GFP positive cells were re-plated in 6 well dishes and grown until confluent, then pools were diluted for selection of single colonies on 10 cm plates where colonies were picked using the cloning cylinder method when viable visible clusters appeared [74]. The viable colonies were grown up and screened via cDNA sequencing and p53 protein analysis.

### Sequence analysis of CRISPR clones

Analysis of CRISPR-Cas9 mediated *TP53* gene editing products within isolated MDA-MB-468 CRISPR clones was performed by Sanger sequencing (GENEWIZ) of PCR amplified cDNA prepared from total RNA isolated from each clone. 5 μg of RNA prepared using the Qiagen RNeasy kit was used for each cDNA synthesis reaction (Applied Biosystems High-Capacity cDNA Reverse Transcription Kit), and the *TP53* gene was subsequently PCR amplified (Phusion polymerase; New England Biolabs) using 5% of the cDNA synthesis product as template and *TP53* gene primers Exon 9–10 Forward and Exon 10–11 Reverse. Sequencing data was generated from cDNA produced from three independent RNA preparations. Sequence analysis of the entire *TP53* gene within parental MDA-MB-468 and MDA-MB-468 CRISPR-Cas9 *CTD sgRNA* clones C11 and C14 was performed by PCR amplification of the *TP53* gene from cDNA using TP53 primers 134–153 5′ Forward (maps to nucleotides 134–153 within Exon1) and 1422–1441 3′ Reverse (maps to nucleotides 1422–1441 within Exon 11). The following primers were used for sequencing *TP53* PCR products: Exon 1F 5′ GAC ACG CTT CCC TGG ATT G; Exon 4R 5′G GGA CAG AAC GTT GTT TTC AGG; Exon 5R 5′ TGT GGA ATC AAC CCA CAG C; Exon 8F 5′ ACA GCA CAT GAC GGA GGT TGT; Exon 8R 5′ CTT GCG GAG ATT CTC TTC CTC; Exon 11R 5′ AGCAAGGGTTCAAAGACCCA.

### Cell culture and cell synchronization

MDA-MB-468 cells and CRISPR derivatives were cultured in complete media (Dulbecco’s Modified Eagle Medium with 4.5g/L glucose containing 10% fetal bovine serum, 50 μg/ml penicillin/streptomycin) at 37°C with 5% CO_2_ and passaged by trypsinization and dilution. Cell cycle synchronization at late G1/early S-phase was achieved using either 5 μM Aphidicolin (ApexBio; 5 mM stock prepared in DMSO), 2 mM Thymidine (Sigma; 100 mM stock prepared in ddH_2_O) or 2 mM Hydroxyurea (Sigma; 200 mM stock prepared in ddH_2_O) by addition of the inhibitor diluted in fresh complete media to 50% confluent cultures grown on 6 cm tissue culture dishes. After a 24-hour incubation with the inhibitor, cultures were either harvested or washed once with Phosphate Buffer Saline (PBS) before addition of fresh complete media to allow cell populations to progress through cell cycle for the indicated time periods.

### Cell cycle analysis

The distribution of cell cultures within G1, S, and G2/M phases was determined by measuring within cell populations the total DNA content relative to cell size after staining chromosomal DNA with Propidium Iodide (PI) using flow cytometry. Cell populations (50–60% confluent) grown in 6 cm tissue culture dishes were harvested by trypsinization, pelleted (low speed-centrifugation in an Eppendorf 5810R centrifuge with a swinging bucket rotor) at 4°C for 7 min. at 500 × *g*, washed with 5 ml of ice-cold PBS, re-pelleted, and resuspended in 1 ml of ice-cold PBS. Cell samples were next fixed in 70% ethanol by the dropwise addition of cell suspensions to 2.5 ml of ice-cold absolute ethanol while vortexing, and subsequently stored at –20°C overnight. After fixation, cell samples were pelleted at 4°C for 7 min. at 700 × *g*, washed twice with 2.5 ml PBS, and then incubated in 0.5 ml PI staining solution (PBS containing 0.1% triton X-100, 200 μg/ml RNaseA, 40 μg/ml propidium iodide) at 37°C for 15 minutes. Following staining, cell samples were filtered through a nylon mesh into polystyrene tubes, and then analyzed on a BD^™^ FACSCalibur instrument. Minimally 10,000 events were counted for each sample and acquired data was analyzed using BD^™^ CellQuest or FlowJo software.

### 5-Bromo-2′-deoxyuridine (BrdU) labeling and measurement of BrdU incorporation by flow cytometry

Cell cultures were pulse labeled with 50 μM BrdU (Sigma; 50mM stock prepared in ddH_2_O) for 30 min, and BrdU incorporated into chromosomal DNA was detected using an Alexa Fluor 488-conjugated anti-BrdU antibody by flow cytometry. Briefly, after labeling with BrdU, cell cultures were harvested, fixed in 70% Ethanol, and stored at –20°C. Following fixation, cells were pelleted, and chromosomal DNA was denatured for 30 min. at room temperature by resuspending cells in 2 ml of Denaturation Solution (2N HCl+0.5% Triton X-100). Post-denaturation, cells were pelleted, neutralized for 10 min at room temperature by resuspension in 2 ml of 0.1M Sodium tetraborate, re-pelleted and then washed once by resuspension in 1 ml of 1% BSA (Bovine Serum Albumin), 0.5% Tween-20 in PBS (Blocking Solution, BLS) before incubation of resuspended cells in 100 μl BLS 1 μg/ml α-BrdU Alexa Fluor 488 with gentle agitation for 2 hours at room temperature. Following the antibody binding step, cells were harvested by pelleting at 1000 rpm for 30 sec. at 4°C, washed three times by resuspension of cell pellets twice with 500 μl blocking buffer and once with PBS containing 0.1% triton-X 100, and then stained with PI staining solution as described above.

### RNA analysis

Total RNA was isolated from cell cultures as dictated by the experimental design using the Qiagen RNeasy kit as directed by the manufacturers (http://www.qiagen.com/HB-0435). The Applied Biosystems High-Capacity cDNA Reverse Transcription kit was used to generate cDNA from 5 μg of total RNA from each cell sample, and the relative abundance of the *TP53,* *RRM2* and *CDC7* mRNA within each was measured by quantitative-PCR using the Applied Biosystems TaqMan Assay with FAM dye-labeled probes (ThermoFisher Scientific *TP53* ID# 01034249_m1, *RRM2* cat# 01072069_g1, *CDC7* ID# 00177487_m1, and *GAPDH* ID# 02786624_g1) and the Applied Biosystems QuantStudio 7 Flex instrument. The relative mRNA expression of *TP53* was also measured with the SYBR green qPCR assay and the primers: exon 9–10 F1 5’TTCACCCTTCAGATCCGTGG and exon 11 R5 5’AGCAAGGGTTCAAAGACCCA.

### Cell extract preparation, SDS-polyacrylamide gel electrophoresis (SDS-PAGE), and western blotting

Total cell lysates were prepared using either 300 mM NaCl RIPA buffer (0. 1% SDS, 1% NP-40, 300 mM NaCl, 1 mM EDTA, 0.5 mM EGTA, 50 mM Tris-Cl pH 8) with 1 mM PMSF, 8.5 μg/ml Aprotinin and 2 μg/ml Leupeptin following standard protocol or Buffer B (50 mM KPO_4_, 1 mM DTT, 1 mM EDTA, 7 mM CHAPS, 10% glycerol, 300 mM NaCl), with each supplemented immediately before use with the following phosphatase inhibitors- 50 mM NaF, 50 μM NaV, and 10 mM β-glycerolphosphate), and protease inhibitors- 0.1 mM PMSF, 1μg/ml Leupeptin, 1 μg/ml Pepstatin A, and 2 μg/ml Aprotinin to achieve the indicated concentrations. Cell cultures were harvested by either trypsinization or scraping using a rubber policeman, and cell populations pelleted at 4°C for 7 min. at 500 × *g*, washed by resuspending with 5 ml of ice-cold PBS, re-pelleted, and then resuspended in ice-cold cell lysis buffer (1 × 10^6^ cells/100 μl buffer). After incubation on ice for 30 min., cell lysates were clarified by centrifugation at 10,000 rpm at 4°C for 20 min, and the protein concentration within each determined using the Bio-Rad Bradford protein assay using BSA as a standard; 10 μg of total protein from each cell lysate was subject to SDS-PAGE (30% Acrylamide:0.4% Bis-acrylamide). Proteins were transferred from each gel onto nitrocellulose membrane and the non-specific binding sites on the resulting western blots were blocked with 1x Blotto (10 mM Tris pH 7.4, 150 mM NaCl, and 3% non-fat dry milk) before probing for the following proteins using the indicated antibodies: p53 (Proteintech cat# ; 1:1000–15,000), RPA (Stillman Laboratory RFA34; 1:1000), PCNA (Novus Biochemicals cat# ; 1:2000), MCM2 (Cell Signaling Technology cat# ; 1:10,000), Lamin A (Sigma cat# ; 1:10,000) RRM2 (Santa Cruz Biotech cat# ; 1:1000), cdc7 (Santa Cruz Biotech cat# ; 1:1000), Cyclin A (Santa Cruz Biotech cat# ; 1:1000), Cyclin B (Santa Cruz Biotech cat# ; 1:1000), and HRP-conjugated Actin (Santa Cruz cat# 47778); All antibodies were diluted in 0.5× Blotto except for RFA34 (1× Blotto was the diluent). Primary antibodies were detected by chemiluminescence using HRP-conjugated anti-rabbit (p53, and Lamin A) or anti-mouse (RPA, PCNA, RRM2, cdc7, Cyclin A, and Cyclin B) secondary antibodies and the Pierce Super Signal^™^ West Pico detection system.

### Glutaraldehyde chemical crosslinking assay

This assay was carried out as described previously for MDA-MB-468 parentals and the CRISPR-cas9 R273H-dual mutants were cultured until 70% confluent [18, 33]. Cells were washed with cold PBS and harvested by scraping in cold PBS and centrifuging at 1100 rpm for 7 min. Harvested cells were lysed with phosphate buffer (PBS, 10% glycerol, 10 mMEDTA, 0.5% NP-40, 0.1M KCl, 1 mM PMSF, 8.5 μg/mL aprotinin, 2 μg/mL leupeptin, and phosphatase inhibitor cocktail). Glutaraldehyde was added to 100 μg of protein lysate to final concentrations of 0.0025 or 0.005%. The lysates were then incubated at room temperature for 20 min with shaking. The crosslinking was stopped by addition of 2X protein sample buffer (2X SDS Laemmli sample buffer, 0.2 M DTT) and heated at 95^o^C for 10 min. 25 μg was resolved on an 8% SDS-PAGE gel and western blot analysis performed with anti-p53 antibody (Santa Cruz, DO-1).

### Cell proliferation assay

Cells were seeded at a density of 1 × 10^5^ cells in 5 ml DMEM complete media on a 6 cm plate and grown for 5 consecutive days. At the end of each 24 hr cycle, cells were washed with PBS followed by trypsinization and counted using a hemocytometer.

### Chromatin fractionation assay

Localization of mtp53 proteins to chromosomes was assessed using two variable abridged versions of the Stillman Chromatin Fractionation Assay [75]. Figure 2 results were derived from the protocol exactly as described previously [20, 75]. Figure 6 cell cycle results had the following revisions, cell cultures (one 50–60% confluent 10 cm plate/sample) as dictated by experimental conditions were harvested by scraping, pelleted and washed with ice-cold PBS, and then resuspended in Buffer A (10 mM HEPES, 10 mM KCl, 1.5 mM MgCl_2_, 300 mM Sucrose, 1 mM DTT, 10% Glycerol, 0.1 mM PMSF, 1μg/ml Leupeptin, 1 μg/ml Pepstatin A, and 2 μg/ml Aprotinin) in a volume of 300 μl (cell density ~1 × 10^7^ cells/ml). Cell populations were lysed by addition of Triton X-100 to a final concentration of 0.15%, incubated on ice for 15 min, and nuclei were separated from the cytosol by centrifugation at 4°C for 4 min at 1,300 × *g*. The resulting cytosolic extract (S1) was collected, and the protein concentration in each sample determined by the Bio-Rad Bradford Assay. The nuclei from each sample were washed twice in 300 μl Buffer A + 0.15% Triton X-100, followed by lysis in 300 μl of Buffer B (3 mM EDTA, 0.2 mM EGTA, 1 mM DTT, 0.1 mM PMSF, 1 μg/ml Leupeptin, 1 μg/ml Pepstatin A, and 2 μg/ml Aprotinin) on ice for 30 min. The chromatin for each sample was separated from the nuclear lysate (S3) by centrifugation for 4 min at 1,700 × *g* at 4°C, washed in 500 μl of Buffer B and collected by centrifugation as described above. The chromatin pellet was resuspended in 1x SDS-PAGE sample buffer, heated to 95°C to denature proteins, and then sonicated on ice to shear genomic DNA. Both the cytosolic fraction (S1) and chromatin pellet for each sample were analyzed by SDS-PAGE and western blotting for the indicated proteins; 10 μg of total protein for each S1 (cytosol) fraction, and a volume corresponding to each S1 was loaded for each chromatin fraction. Comparable recovery of chromatin from each sample was confirmed by detection of all four core histones on Ponceau S-stained membranes.

## SUPPLEMENTARY MATERIALS



## Abbreviations

TNBC: Triple negative breast cancer
GOF: Gain-of-function
Mtp53: Mutant p53
CTD: C-terminal domain
OD: Oligomerization domain
RRM2: Ribonuclease Reductase Member 2
MCM2: Minichromosome Maintenance 2
PCNA: Proliferating Cell Nuclear Antigen
