# Supplementary material for: Frame-shift mediated reduction of gain-of-function p53 R273H and deletion of the R273H C-terminus in breast cancer cells result in replication-stress sensitivity
Source: Oncotarget. 2021 Jun 8;12(12):1128–46. doi: 10.18632/oncotarget.27975 (PMC8202772; doi:10.18632/oncotarget.27975)
Supplement: Supplementary file 1 [file oncotarget-12-1128-s001.pdf]

Frame-shift mediated reduction of gain-of-function p53 R273H and deletion of the R273H C-terminus in breast cancer cells result in replication-stress sensitivity

SUPPLEMENTARY MATERIALS

```
CRISPR-C14      ------------------------------QIRGRERFEMFRELNEALELKDAQXGKEPG
CRISPR-C4       ------------------------------QIRGRERFEMFRELNEALELKDAQAGKEPG
TP53            ------------------------------QIRGRERFEMFRELNEALELKDAQAGKEPG
CRISPR-C13      ------------------------------QIRGRERFEMFRELNEALELKDAQAGKEPG
CRISPR-C5       ------------------------------QIRGRERFEMFRELNEALELKDAQAGKEPG
CRISPR-C1       ------------------------------QIRGRERFEMFRELNEALELKDAQAGKEPG
CRISPR-C11      ------------------------------QIRGRERFEMFRELNEALELKDAQAGKEPG

CRISPR-C14      GSRAHSSHLKSKKGQSTSRQ--------GPDSD--------------
CRISPR-C4       GSRAHSSHLKSKKGQSTSRHKKLMFKKGLTQTDILHF-CPHXQH---
TP53            GSRAHSSHLKSKKGQSTSRHKKLMFKTEGPDSD--------------
CRISPR-C13      GSRAHSSHLKSKKGQSTSRHKKLMFKKGLTQTDILHF-CPHXQXXXX
CRISPR-C5       GSRAHSSHLKSKKGQSTSRHKKLMFKKGLTQTDILHFLSPLTAXXXX
CRISPR-C1       GSRAHSSHLKSKKGQSTSRHKKLMFKKGLTQTDILHFLSPXTAXXXX
CRISPR-C11      GSRAHSSHLKSKKGQSTSRHKKLMFKKGLTQTDILHFLFPTDSLPPSL
```

Supplementary Figure 1: Sequence analysis of MDA-MB-468 CRISPR-Cas9 generated mtp53 variant-expressing cell lines. The sequence of the TP53 gene encompassing Exons 10 and 11 from cell clones C1, C4, C5, C11, C14, and C14 derived from *C-REG sgRNA* CRISPR-Cas9 modified MDA-MB-468 breast cancer cells was determined by Sanger sequencing of PCR amplified cDNA generated from random primed total RNA isolated from each cell line. Shown is an alignment of the translated sequence of each with that of the MDA-MB-468 *TP53* reference sequence created using the MUSCLE 3.8 CLUSTAL multiple sequence alignment tool. Sequencing data was generated from cDNA produced from 3 independent RNA preparations of each cell line, and for clones C11 and C14 the entire *TP53* gene was sequenced and no additional changes were found.

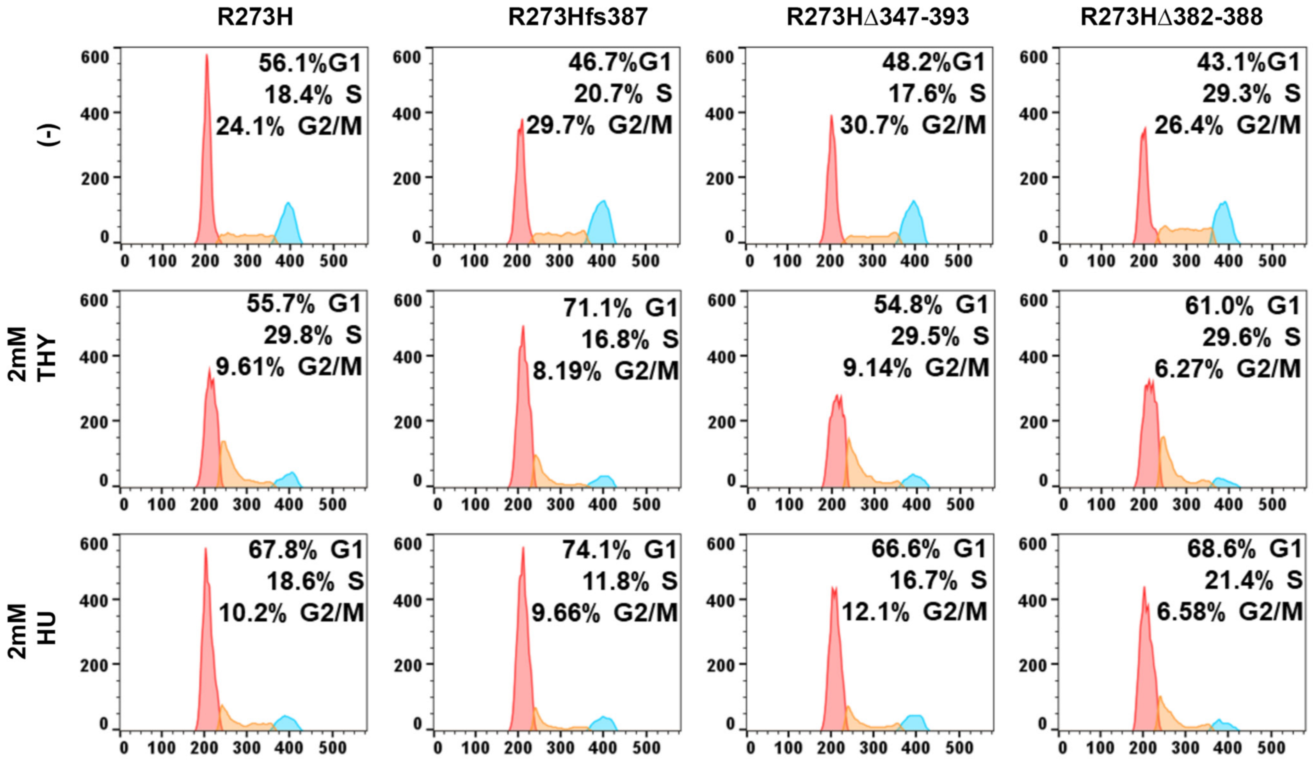

Supplementary Figure 2: Correlation of mtp53 variants and RRM2 protein abundance with reduced *TP53* mRNA levels in MDA-MB-468 CRISPR-Cas9 generated mtp53 variant-expressing cell lines. Sub-confluent cultures (~50% confluent) of parental MDA-MB-468 mtp53+ (R273H) and CRIPSR-generated mtp53 variants cell lines mtp53-depleted (R273H*fs*387), mtp53Δ47 (R273HΔ347), and mtp53Δ8 (R273HΔ381-388) were synchronized at G1/S by treatment of cell populations with either 2 mM thymidine (Thy) or 2 mM hydroxyurea (HU) for 24 hours, and then harvested and split into three aliquots that were processed for flow cytometry and the analyses presented in Figure 4 as described in the Materials and Methods. Presented within each histogram of the indicated the cell population (asynchronous (–) or G1/S synchronized) is the percentage of cells within G1, S and G2/M.

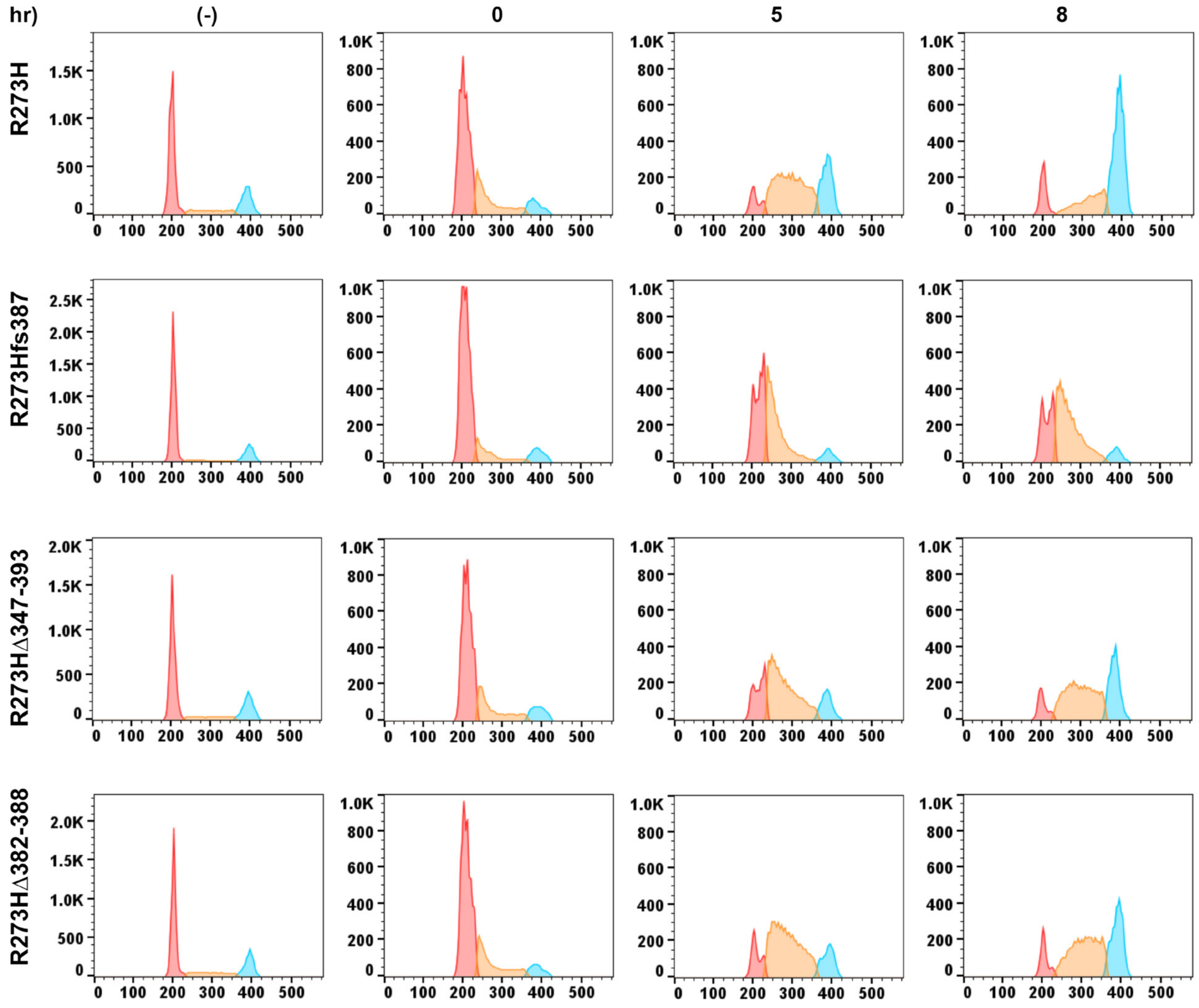

Supplementary Figure 3: MDA-MB-468 CRISPR-Cas9 generated mtp53 variant-expressing cells display thymidine sensitivity characterized by slow progression through S-phase. The kinetics of S-phase progression of parental and CRISPR variant cell lines (mtp53+, mtp53-depleted, mtp53Δ47, and mtp53Δ8) were compared post synchronization of 50% confluent cultures of each with 2 mM thymidine. At time points 0, 5, and 8 hours post release from the Thy block, cell populations from each cell line were harvested simultaneously and the cell cycle distribution of propidium iodide-stained cells was determined by flow cytometry as described in the Materials and Methods. The distribution of cells within the G1, S, and G2/M phases at each time point within each population correspond to the super-imposed histograms represented in Figure 5A of mtp53+ cells (R273H yellow), mtp53-depleted cells (R273H*f*s387 gray), mtp53Δ47 cells (R273HΔ347 red), and mtp53Δ8 cells (R273HΔ381-388 green).

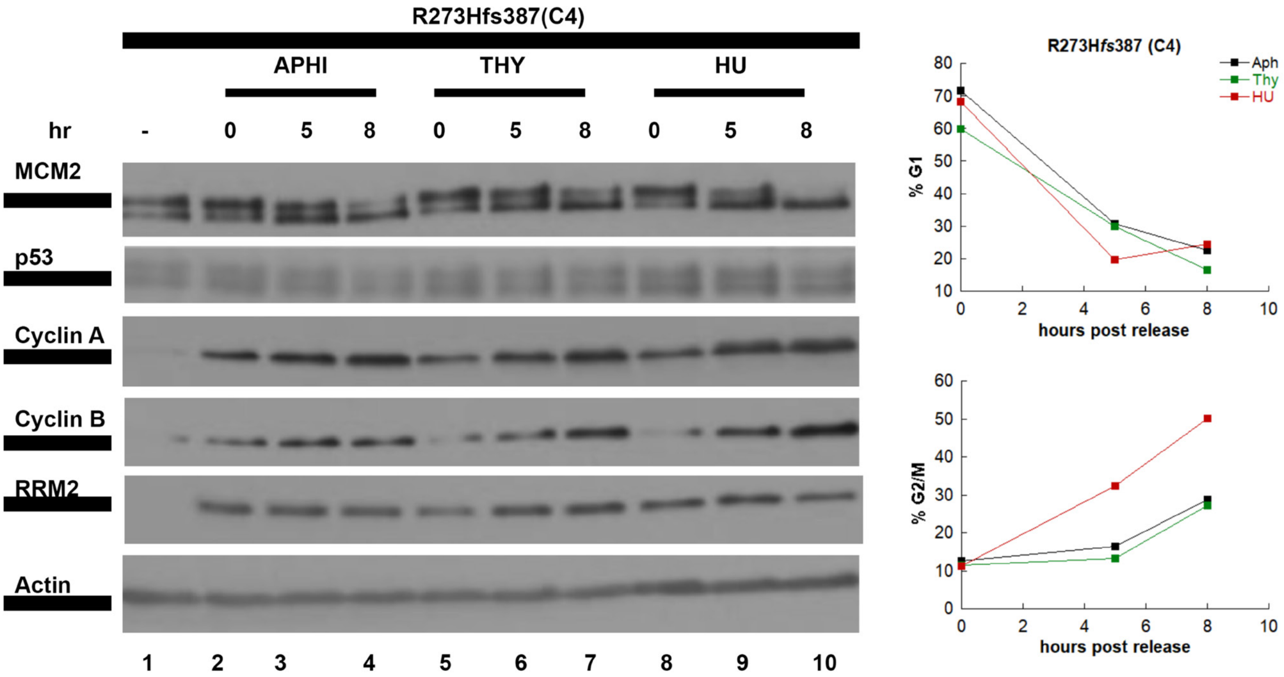

Supplementary Figure 4: MDA-MB-468 CRISPR-Cas9 generated mtp53 variant-expressing cells display thymidine sensitivity characterized by slow progression through S-phase. The abundance of the p53, MCM2 and RRM2 proteins were examined in extracts from asynchronous (–) and G1/S synchronized cell populations of the MDA-MB-468 CRIPSR-generated mtp53-depleted (R273H*fs*387) variant cell line C4 harvested 0, 5, and 8 hours post release from a 24 incubation with the cell cycle inhibitors aphidicolin (Aph), Thymidine (Thy), or hydroxyurea (HU). Cells were cultured to 50% confluency before addition of either 5 µM Aph, 2 mM Thy, or 2 mM HU, and at the above time points cell populations were harvested and processed for either cell cycle analysis by flow cytometry, or western blotting. The distribution of cells within G1, S, and G2 based on propidium iodine (PI) staining was determined as described in the Materials and Methods, and the percentage within G1 and G2 for each time point is represented in the graphs on the right.

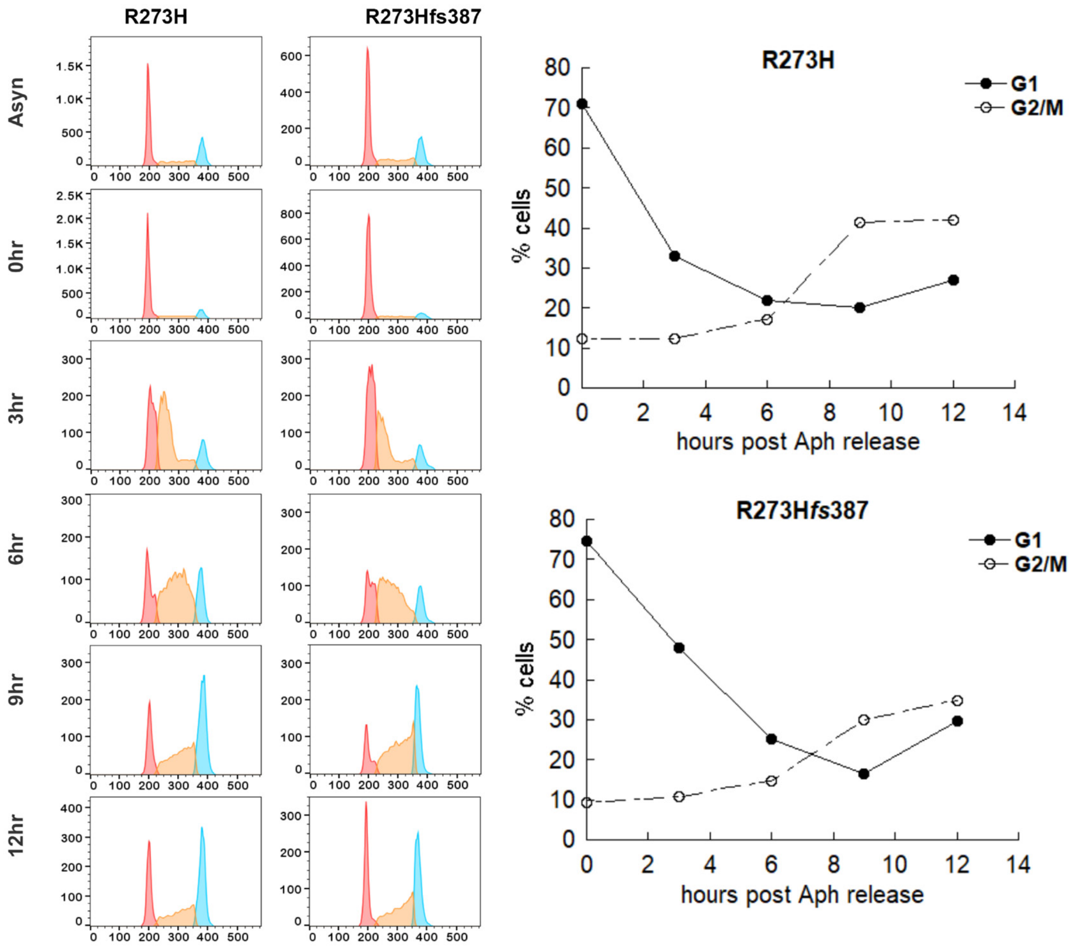

Supplementary Figure 5: MDA-MB-468 CRISPR-Cas9 generated mtp53 variant-expressing cells display comparable kinetics of S-phase progression through S-phase when synchronized with Aphidicolin. The kinetics of S-phase progression of parental MDA-MB-468 mtp53+ (R273H) and CRIPSR-generated mtp53-depleted (R273H*fs*387) variant cell line C11 were compared post synchronization of 50% confluent cultures with 5 µM Aphidicolin. At time points 0, 3, 6, 9, and 12 hours post release from the Aph block, cell populations from each cell line were harvested simultaneously and the cell cycle distribution of propidium iodide-stained cells was determined by flow cytometry as described in the Materials and Methods. The percentage of cells within G1 and G2 presented in the histograms for each cell population is represented in the graphs for each cell line on the right.
